# A Nitrifying Bacteria-Based Oxygen Consumption Assay for Multifaceted Soil Toxicity Monitoring

**DOI:** 10.3390/toxics13110937

**Published:** 2025-10-30

**Authors:** Suleman Shahzad, Aparna Sharma, Syed Ejaz Hussain Mehdi, Fida Hussain, Sandesh Pandey, Mudassar Hussain, Woochang Kang, Sang Eun Oh

**Affiliations:** 1 Department of Biological Environmental, Kangwon National University, Hyoja-2-dong, Chuncheon-si 24341, Gangwon-do, Republic of Korea; suleman@kangwon.ac.kr (S.S.); aparna06@kangwon.ac.kr (A.S.); syedejaz@kangwon.ac.kr (S.E.H.M.); hussainfida@korea.ac.kr (F.H.); sandeshpandey@kangwon.ac.kr (S.P.); ajakwc@kangwon.ac.kr (W.K.); 2 Department of Environmental Science, University of Lahore, Lahore 545590, Pakistan; 3 Department of Pathology and Microbiology, Division of Microbiology, School of Medicine, Nihon University, Itabashi-ku, Tokyo 173-8610, Japan; hussain.mudassar@nihon-u.ac.jp

**Keywords:** soil toxicity, nitrifying bacteria, bioassays, harmful elements, toxicity threshold, soil health, field assessment

## Abstract

Soil toxicity resulting from either natural or anthropogenic heavy metal contamination was evaluated through a nitrifying bacteria bioassay focused on the inhibition of oxygen consumption. Every contaminated soil sample inhibited the nitrifying bacteria bioassay, with inhibition levels ranging from 71% to 100%. The optimal conditions for maximizing O_2_ consumption during the test procedure were established as follows: a test culture volume of 1 mL, a soil sample weight of 1 g, a rotation rate of 100 revolutions per minute, and a reaction duration of 48 h. In low- or uncontaminated soils, oxygen consumption ranged from 3.2 mL to 3.0 mL from a headspace volume of 1 mL filled with O_2_. In contrast, contaminated soils exhibited a lower range, with values between 0.1 mL and 1.0 mL. EC50 levels for NB O_2_ consumption were: Cr^6+^ 1.21 mg/kg; Cu^2+^ 6.92 mg/kg; Ag^+^ 8.38 mg/kg; As^3+^ 8.99 mg/kg; Ni^2+^ 10.35 mg/kg; Hg^2+^ 11.01 mg/kg; Cd^2+^ 31.33 mg/kg; Pb^2+^ 129.62 mg/kg. Values for inherent test variability (CVi), variation resulting from the natural characteristics of soil (CVns), and minimal detectable difference (MDD) were found to range between 1.6% and 4.7%, 7.8% and 14.6%, and 2.9% and 5.9%, respectively. A 10% toxicity threshold was set as the maximal tolerable inhibition (MTI) for effective soil toxicity assessment. Nitrifying bacteria bioassays offer a fast, affordable, and user-friendly tool for real-time soil toxicity assessment, boosting soil health monitoring and ecosystem protection.

## 1. Introduction

The pollution caused by harmful elements (HMs), mainly due to extensive mineral extraction and usage, has emerged as a significant global concern [1]. Microbial bioassays allow surveillance of pollutants not easily captured by standard analytical methods. Bioassays offer a clear and effective way to measure the cumulative and synergistic toxicity of chemical mixtures, surpassing traditional methods that focus on individual toxicants [2,3].

The complexity and variability of soil texture, organic matter, and particle size create sorption sites for contaminants, which in turn limit the bioavailability of toxicants to the organisms being tested [3,4]. Prior toxicity bioassays involving organisms, such as *Vibrio fischeri*, *Selenastrum capricornutum*, *Pseudomonas putida*, and *Photobacterium phosphoreum*, were employed to evaluate detrimental elements, organic contaminants, and biohazardous compounds present in polluted soils. Nonetheless, these methods have typically been restricted to evaluating the reactions of microorganisms subjected solely to soil extracts (elutriates) that are prepared in either water or organic solvents [5,6].

Elutriation often disrupts the natural interactions between microorganisms and the solid phase of contaminated soils, leading to inaccurate estimates of toxicity. The adsorptive and textural properties of soils—particularly those rich in clay and silt—can reduce contaminant bioavailability, cause variable permeability, and introduce measurement inconsistencies in elutriate-based microbial assays [7,8,9,10]. To overcome these limitations, direct-contact bioassays have been developed, allowing microorganisms to interact directly with unmodified solids and thereby providing a more realistic, solvent-free evaluation of soil toxicity [2,3,11].

In this study, employing a nitrifying bacteria-based direct-contact bioassay offers additional ecological relevance, as these bacteria are native to soil and play a key role in the nitrogen cycle. Their sensitivity to pollutants provides meaningful insights into how contaminants disrupt fundamental soil processes and microbial functionality [12,13,14,15]. Consequently, this approach enables a more comprehensive assessment of soil health and pollutant impacts than conventional liquid-phase bioassays, which remain more widely used despite their methodological limitations [16,17].

2 NH_4_^+^ + 3 O_2_ → 2 NO_2_^−^ + 2H_2_O + 4H^+^ (1)

2 NO_2_^−^ + O_2_ → 2 NO_3_^−^ (2)

Heavy metal levels in contaminated soils vary with source and land use, typically ranging from Ag^+^ (1–50 mg kg^−1^), As^3+^ (5–500 mg kg^−1^), Cd^2+^ (1–100 mg kg^−1^), Cu^2+^ (10–1000 mg kg^−1^), Cr^6+^ (5–800 mg kg^−1^), Hg^2+^ (0.1–30 mg kg^−1^), Ni^2+^ (5–700 mg kg^−1^), and Pb^2+^ (10–2000 mg kg^−1^). In contrast, background concentrations in uncontaminated Korean soils are much lower (e.g., Cd ≈ 0.3 mg kg^−1^, Cu ≈ 15 mg kg^−1^, Pb ≈ 18 mg kg^−1^, Cr ≈ 25 mg kg^−1^), indicating significant enrichment near industrial and mining areas [18]. Since the bacteria are native to the soil environment, the bioassay conditions closely mimic natural conditions, leading to results that are more representative of real-world scenarios [12,19]. Nitrifying bacteria bioassays are advantageous for soils with high clay and silt content, as they target bacteria naturally involved in the nitrogen cycle, offering a more ecologically relevant assessment of contamination [19,20]. These bioassays can better reflect the actual impact on microbial processes, despite the reduced bioavailability of contaminants in such soils [21]. Essential genes, including *amoA* from ammonia-oxidizing archaea (AOA), ammonia-oxidizing bacteria (AOB), and complete ammonia-oxidizing bacteria (CAOB), encode the enzyme ammonia monooxygenase, which drives the conversion of ammonia to nitrite. Following this, nitrite-oxidizing bacteria (NOB) carrying the *nxrA* and *nxrB* genes, which code for nitrite oxidoreductase, facilitate the oxidation of nitrite into nitrate [22,23].

The aims of this research were: (i) to create a bioassay for nitrifying bacteria in direct contact to evaluate soil toxicity through the measurement of oxygen consumption; (ii) to enhance essential testing parameters for consistent and repeatable performance; (iii) to ascertain the EC_50_ values for significant toxic elements (Ag^+^, As^3+^, Cd^2+^, Cu^2+^, Cr^6+^, Hg^2+^, Ni^2+^, and Pb^2+^) through the optimized assay; and (iv) to implement the specified toxicity threshold in the evaluation of naturally contaminated soils originating from various industrial sites in South Korea. This study also aimed to highlight the novelty and ecological relevance of using nitrifying bacteria as sensitive indicators of soil health, offering a swift, field-applicable, and environmentally realistic option compared to conventional elutriate-based bioassays.

## 2. Materials and Methods

### 2.1. Ammonia-Based Nitrifying Bacteria Master-Culture Reactor (NBMCR)

**Figure 1:**
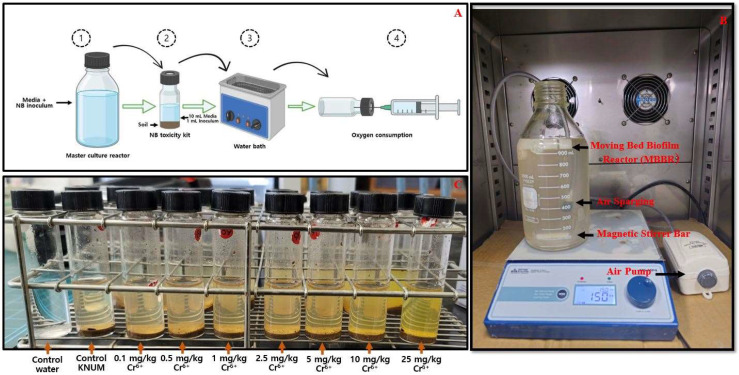
(**A**) Schematic diagram, (**B**) picture of nitrifying bacteria master culture reactor (NBMCR) cultivation, (**C**) NB soil toxicity test kit.

A schematic diagram illustrated the nitrifying bacteria bioassay (Figure 1A). A 1 L master culture reactor (NBMCR) for aerobic nitrifying bacteria was established to provide consistently uniform nitrifying bacterial cultures. In accordance with our prior investigation (Shahzad et al., 2025) [24], the nitrifying bacteria reactor was inoculated with aerobic return-activated sludge obtained from the Chuncheon Wastewater Treatment Plant, Republic of Korea. This study applied the same enrichment and operational conditions to ensure the consistency and reproducibility of the bacterial culture utilized for soil toxicity testing. According to a recent report (Shahzad et al., 2025) [24], the basal mineral media (BMM) for nitrifier enrichment contains (per L): 0.38 g NH_4_Cl, 0.31 g KH_2_PO_4_, 0.02 g CaCl_2_·2H_2_O, 0.04 g MgSO_4_·7H_2_O, 0.1 mg NaMoO_4_·2H_2_O, 0.2 mg MnCl_2_, 0.002 mg CoCl_2_·6H_2_O, and 0.1 mg ZnSO_4_·7H_2_O. The NBMCR continuously stirred at 100 RPM and operated in a fill-and-draw mode, with 250 mL removed and replaced with 250 mL of fresh media every three days (Figure 1B). The reactor temperature was kept constant at 30 °C, and the initial pH was 7.8 (Figure S1). After sterilization, a 0.5 M sodium carbonate solution was added to control pH, and readings were taken using a LUTRON pH-208c pH meter. Ammonia, nitrate, and nitrite values were monitored using ion chromatography in NBMCR (Figures S2–S4). The method for measuring ion chromatography was explained in (S1:M1). Before its use in batch mode was tested, the NBMCR operated in fill-and-draw mode for over nine months.

### 2.2. Soil Sampling and Characterization

Eleven uncontaminated reference soils (Table 1), which include the mountain soil from Kangwon National University (KNUMS), were utilized as controls in this study. In addition, nine naturally contaminated field soils (Table 2) were collected from the 0–30 cm surface layer at the specified sampling sites. KNUMS was employed as a negative control. At each site of sampling, plant roots, debris, leaves, and coarse materials were gathered, thoroughly hand-mixed, placed in plastic bags, and conveyed to the laboratory for further processing and analysis. Soil samples were filtered using 2 mm sieves and then stored in polystyrene bottles with screw-on lids at ambient temperature before being employed in toxicity testing. The analysis of soil texture, which includes the proportions of sand, silt, and clay, was conducted using the pipette method as described by [24]. The organic matter content was further determined by loss-on-ignition at 550 °C in a muffle furnace. Soil pH and electrical conductivity (EC) were evaluated with an Orion Versa Star Pro multi-parameter benchtop meter at a soil-to-liquid ratio of 1:5. Background levels of harmful elements were quantified by reverse aqua regia digestion following USEPA (1986) and [25], with analysis performed on an ICP-OES (Optima DV, PerkinElmer, Waltham, MA, USA). The measurement of total petroleum hydrocarbons (TPH) was carried out in accordance with the methodology established by [26]. For accurate quantification of heavy metals and organic contaminants in the soil samples, each digestion batch incorporated National Institute of Standards and Technology (NIST) certified reference material SRM 2711a (Montana II Soil) at a 5% inclusion rate. The resulting accuracy, expressed as percent recovery, was maintained within ± 15%.

### 2.3. Optimization and Toxicity Testing Using Nitrifying Soil Toxicity Kit

The components of the NB soil toxicity kit were altered to ascertain the ideal conditions for assessing soil toxicity during the optimization of the testing phase. The preliminary control tests involved assessing how different soil types and varying mixing intensities (RPM) influenced gas consumption in the NB bioassay. Additionally, supplementary tests were conducted to examine how different soil types influence the O_2_ consumption activity of NB. At this stage, 1 g of each soil type (loam, sand, silt, and clay) was combined with 1 mL of NB inoculum and 9 mL of basal mineral medium in separate 25 mL vials. The samples were incubated at 30 °C with shaking at 100 RPM, resulting in a total working volume of 10 mL. The influence of various mixing speeds (10, 25, 50, and 100 RPM) on the gas consumption of NB was analyzed under the conditions detailed earlier. Under the determined optimized conditions, batch testing was executed to analyze the inhibitory influence of the harmful elements on the O_2_ consumption assay of NB after an exposure duration of 48 h. For this assessment, appropriate volumes of standard heavy-metal toxicant solutions were added to test kits containing 1 g of soil, the test culture, and the working media (Figure 1C). The concentration ranges for the harmful elements examined in this research are outlined in Table 3. Ultimately, the newly developed NB test kit was employed to assess the toxicity of field soils that were “naturally” contaminated, collected from various contaminated hotspots across South Korea. For this experiment, 10 uncontaminated reference soils (Table 1) and nine contaminated field soils (Table 2) were employed. In the experiment, 1 g of contaminated field soil was mixed with 10 mL of working medium and 1 mL of test culture, and then incubated at 30 °C with shaking at 100 RPM for 48 h.

### 2.4. NB Kit Protocol for Soil Toxicity Testing

NB bioassay test kits (Figure 1C) were employed to compare the toxicity of reference soils and contaminated field soils, with particular attention to NB’s oxygen consumption activity. The existing experimental arrangement has undergone slight modifications to the approach previously detailed by [23]. Briefly, 1 g of the soil sample was placed in 25 mL flat-bottom glass vials fitted with plastic caps and Teflon-lined rubber stoppers to retain VOCs from the contaminated soil. Afterward, 9 mL of the modified NMB medium was added to glass vials, which were kept in a water bath at 30 °C. Upon reaching 30 °C in the water bath, each soil–medium vial received 1 mL of the mixed NB culture. The test kit headspace (15 mL) was purged with pure oxygen for one minute and quickly sealed using plastic caps equipped with Teflon-lined rubber stoppers. The NB bioassay test kits, once prepared, were swiftly transferred and positioned horizontally in a stacked arrangement inside a shaking water bath set to a temperature of 30 °C and a speed of 100 rpm. Due to the oscillatory shaking of the water bath, oxygen dissolved more effectively in the headspace. Following 10 min of shaking, the kits were briefly taken from the water bath, and a 26 G needle was passed through the Teflon rubber stoppers for 5 s to equalize internal and external pressure. After achieving pressure equilibration, the test kits were swiftly placed back into the shaking water bath for an additional 48 h of incubation. In addition to the reference and polluted samples, two control setups were used: a negative control with standard soil and a water control without soil. To measure NB activity, the water control was performed together with the reference and contaminated soil tests. At 12 h intervals through 48 h of incubation, oxygen consumption by NB was measured in each test kit using a 10 mL glass syringe lubricated with a solution prepared from 5 drops of detergent in 100 mL distilled water. With lubrication complete, the plunger was set to 10 mL on the syringe. The needle was introduced parallel to the ground through the Teflon stopper into the vial, and the plunger advanced into the barrel until atmospheric pressure equilibrium was achieved. The indicated syringe reading corresponds to the oxygen consumed by NB [23]. The inhibitory effect (percentage) of test-soil toxicants on NB oxygen consumption was derived by comparing the observed decrease in oxygen use in the samples to that of the negative control.

### 2.5. Stoichiometric O_2_ Demand in Test Kits

Using Equations (1) and (2), we calculated the theoretical O_2_ demand for NB. Based on 100 mg/L ammonia utilization and the ideal gas relation (1 mol = 22.4 L at STP), the O_2_ volume at 30 °C was derived, yielding an O_2_ requirement of 3.3 mL for 100 mg/L ammonia.

### 2.6. Quantitative Analysis and Biostatistics

Analytical-grade chemicals, purchased from Sigma-Aldrich (St. Louis, MO, USA), were used throughout without additional purification. To evaluate heavy metal toxicity, we used AgNO_3_, CuSO_4_·5H_2_O, CdSO_4_·8/3 H_2_O, NaAsO_2_, Pb(NO_3_)_2_, HgCl_2_, K_2_Cr_2_O_7_, and NiCl_2_·6H_2_O. Oxygen consumption in the test kits was measured using glass syringes. Each assay run in duplicate, and results reported as mean ± standard deviation

Using Equation (3), we determined inhibition (percentage) as the reduction in gas consumption relative to the negative control to assess how the tested harmful elements affected NB gas consumption activity.

(3) $$Inhibition\;rate\left(\%\right)=\left(1-\frac{Oxygen\;consumption\;in\;test\;samples}{Oxygen\;consumption\;in\;control\;soil}\right)\times 100$$

EC_50_, defined as the concentration at which gas consumption is inhibited by 50%, was determined with Sigma Plot 14.0 (Systat Software Inc., San Jose, CA, USA). Next, Sigma Plot 14.0 was used to construct dose–response curves illustrating how each toxicant inhibited the NB gas-consumption assay. In the same manner, multiple statistical approaches are used to estimate the toxicity threshold, facilitating the evaluation of toxicity in contaminated field soils. For each reference soil, differences in gas consumption were assessed via the coefficient of variation (*CVi*) calculated from Equation (4).

*CV_i_* = *SD_x_*/*Mean_x_* × 100 (4)

The effect of diverse soil physicochemical parameters on NB gas consumption was quantified using the coefficient of soil variation (CV*_ns_*), calculated from the standard deviation and the mean of percent inhibition for each reference soil relative to the negative control (KNU mountain soil), following Equation (5).

CV*_ns_* = SD*_ns_*/Mean*_ns_* × 100 (5)

In addition, the MDD [Equation (6)]—a measure of a test’s ability to discriminate [27]—was derived from the intrinsic variability of NB gas consumption observed for each sample

(6) $$\%MMDx=\frac{100t\sqrt{{\frac{\left(SDc\right)}{nc}}^{2}+{\frac{\left(SDc\right)}{nx}}^{2}}}{Meanx}$$

Ultimately, the Maximal Tolerable Inhibition (MTI), which captures variation in gas consumption among the reference soils, was obtained using Equation (7).

MTI (%) = *Average* (*I*) + *SD* (*I*) (7)

Using Sigma Plot 14.0, we produced inhibition plots for the contaminated soils; plots that crossed the established threshold considered toxic to NB. We applied PCA in R-Studio to clarify the influence of various soil physicochemical characteristics on NB gas production across reference and contaminated soils [28,29]. Spearman’s rank correlation (*p* = 0.05) was employed to examine links between gas consumption and the diverse physicochemical traits of both reference and contaminated soils.

## 3. Result and Discussion

### 3.1. NBMCR Operation

The NBMCR was utilized to enhance, culture, and sustain stable nitrifying bacteria, ensuring consistent outcomes in optimization and toxicity assessments. The reactor displayed a biomass concentration (Figure S3). Under the applied testing conditions and methods, a sustainable nitrifying bacteria community was established, yielding effective nitrification by 48 h post-inoculation. During every feeding and waste-removal cycle, ammonium underwent full oxidation, reducing its concentration. During the course of nitrification, the pH levels began at 7.8 and subsequently decreased to approximately 5.2 upon the completion of the nitrification process (Figure S1). Throughout the process of establishing the baseline condition, there was a gradual decline in pH values over time.

### 3.2. Comparative Physicochemical Analysis of Reference and Contaminated Soils

Table 1 summarizes the physicochemical properties of the reference soils. Of these, eight were classified as sandy loam, and the remainder as sand clay loam. The pH values observed range from 5.6 at WON1 to 7.5 at CHN1, with the electrical conductivity for KNUM measured at 0.5 mS/cm. The analysis revealed that the concentrations of harmful elements, specifically Cd, As, Cu, Ni, Pb, and Zn, in reference soils were minimal, indicating an absence of toxicity potential in the NB bioassay. Table 2 presents the physicochemical analysis of the contaminated soils used in this study. Among them, nine were classified as sandy loam and one as sandy clay. EC ranged from 0.1 mS/cm at UST to 0.6 mS/cm at FMP; pH ranged from 6.2 at GRG to 8.1 at ISF. Concentrations of Ni, As, Cd, Cu, Pb, and Zn were dangerously high and could be toxic to NB.

### 3.3. Optimization of Test Parameters

**Figure 2:**
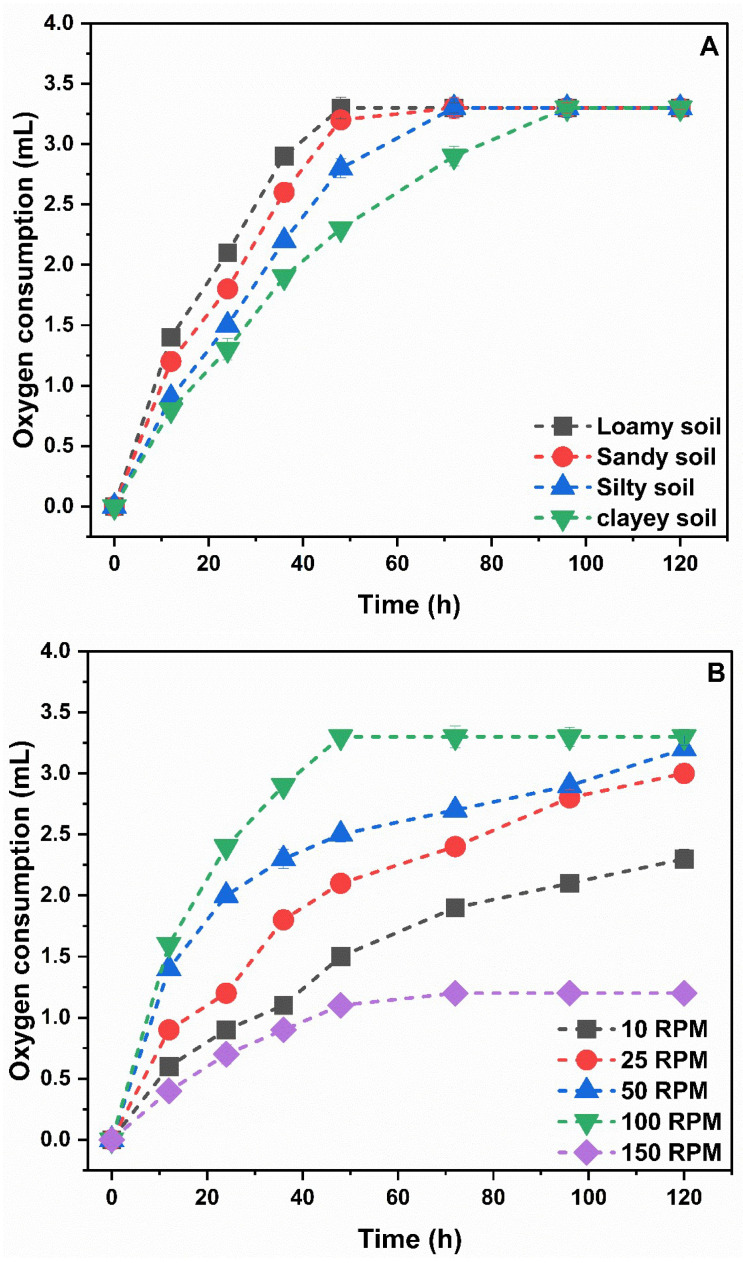
(**A**) Effect of different soil types on gas production by NB, (**B**) effect of increased mixing speed (RPM) on gas consumption by NB.

The evaluation of soil-type effects on NB indicated comparable O_2_ use (3.3 mL) for all four soil types. Gas consumption was rapid and reached completion after 48–72 h in loamy and sandy soils (Figure 2A). On the other hand, the reaction rate in silty and clay soils was slower, resulting in a reaction time that lasted for 72 and 96 h (Figure 2A). Maintaining soils neither desiccated nor waterlogged likely lowered nitrification rates, resulting in a 96 h reaction time. At agitation speeds of 50 and 100 RPM, the NB test kits utilized approximately equivalent O_2_ volumes of about 3.3 mL. Simultaneously, the test kits exhibited a reduction in O_2_ consumption (1.9 mL and 2.4 mL) after being agitated for 72 h at rotational speeds of 10 and 25 RPM, respectively. However, the cessation of O_2_ consumption occurred at different times depending on the RPM. The cessation of gas consumption occurred after only 48 h in the 100-RPM test, in contrast to the 50-RPM test, which required 120 h for gas consumption to stop (Figure 2B). The 150-RPM mixing of soil and NB appears to have broken up the culture, lowering NB activity. On this basis, an optimal duration of 48 h was selected for all tests.

### 3.4. Evaluation of Heavy Metal Toxicity via Nitrifying-Bacteria Oxygen Demand

**Figure 3:**
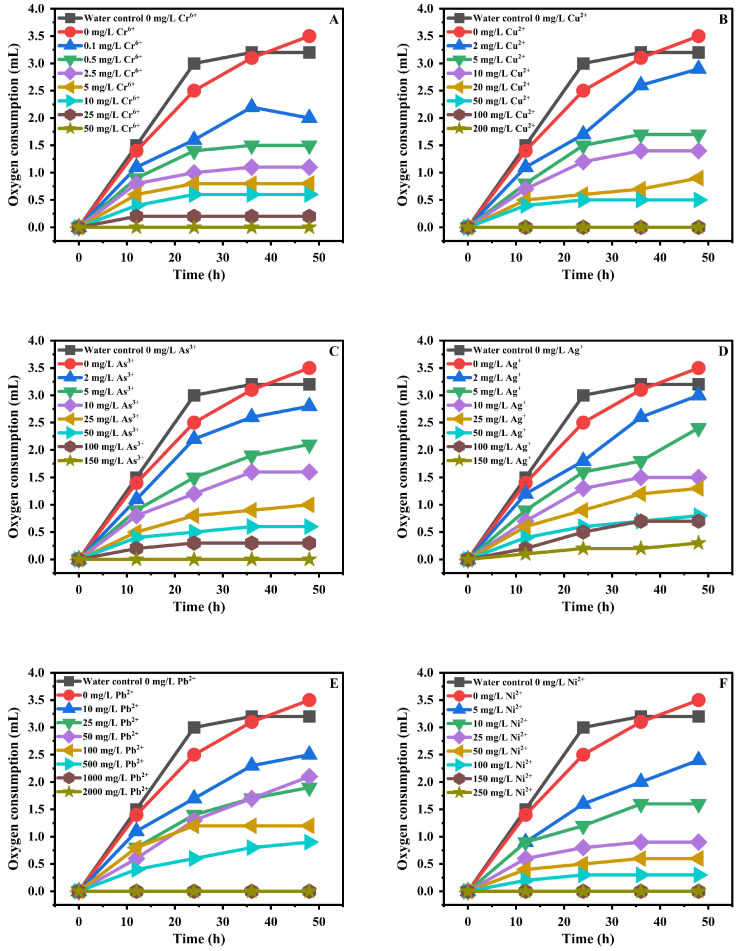

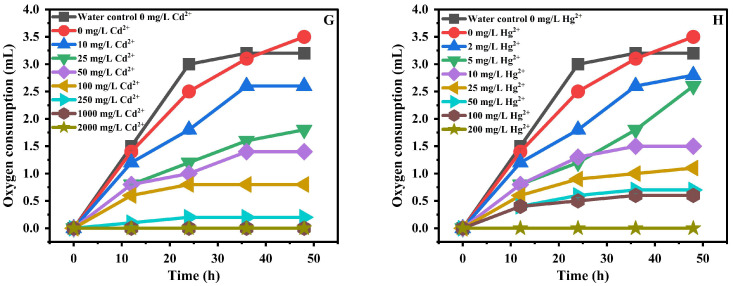
Gas consumption by NB in test soils spiked with varying concentrations of (**A**) Cr^6+^, (**B**) Cu^2+^, (**C**) As^3+^, (**D**) Ag^+^, (**E**) Pb^2+^, (**F**) Ni^2+^, (**G**) Cd^2+^, (**H**) Hg^2+^ into the test soils.

**Figure 4:**
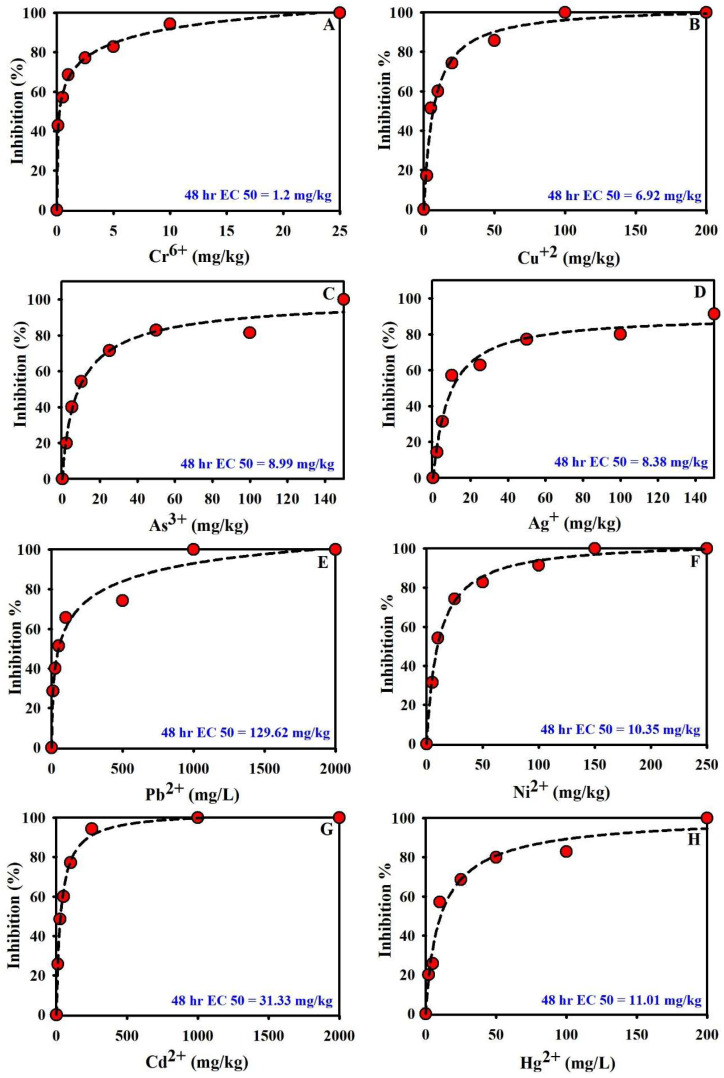
Levels of inhibition of gas consumption activity of NB following the introduction of (**A**) Cr^6+^, (**B**) Cu^2+^, (**C**) As^3+^, (**D**) Ag^+^, (**E**) Pb^2+^, (**F**) Ni^2+^, (**G**) Cd^2+^, (**H**) Hg^2+^ into the test soils.

Figure 3 and Figure 4 display the dose–response relationships from the NB soil toxicity assay using varying concentrations of Ag^+^, Cr^6+^, Cu^2+^, Hg^2+^, Ni^2+^, Pb^2+^, As^3+^, and Cd^2^. In general, increasing toxicant levels intensified NB inhibition and reduced O_2_ consumption. However, various heavy metal species produced distinct patterns, degrees of inhibition, and toxic effects in the NB assay. Notably, Cr^6+^ proved to be highly effective at a dosage of 0.5 mg/kg, whereas increasing the concentration to 25 mg/kg resulted in total inhibition of NB activity, with no O_2_ being consumed. Ni^2+^ exhibited relatively mild toxicity toward NB: a 50 mg/kg dose produced 82.85% inhibition of activity. Similarly, the metals exhibited distinct behaviors when spiked at 5 mg/kg and 100 mg/kg. NB inhibition reached 40% at 5 mg/kg As^3+^ and 85.57% at 100 mg/kg. For 5 mg/kg, Hg^2+^ and Cu^2+^ produced 25.71% and 82.85% inhibition, respectively; at 100 mg/kg, they yielded 51.42% and 100%. Consistent with predictions, the introduction of 200 mg/kg of Ni^2+^, As^3+^, Hg^2+^, and Cu^2+^ into the samples led to a complete halt in NB activity, as reflected by the lack of O_2_ consumption. Conversely, Pb^2+^ and Cd^2+^ exhibited milder effects, with 100 mg/kg yielding 65.71% inhibition for Pb^2+^ and 77.14% for Cd^2+^. The observation of NB’s activity was completely terminated at the 1000 mg/kg level of Cd^2+^ and Pb^2+^. The reason for the negligible inhibition of NB activity at lower concentrations of Cd^2+^ may stem from the lower bioavailability of Cd^2+^ at 5 mg/L as opposed to 10 mg/L, given that a significant quantity of Cd^2+^ precipitated and adsorbed by soil (Figure 4G). In this study, the determined EC_50_ values for the metals tested, listed from lowest to highest, are: 1.2 mg/kg for Cr^6+^; 6.92 mg/kg for Cu^2+^; 8.38 mg/kg for Ag^+^; 8.99 mg/kg for As^3+^; 10.35 mg/kg for Ni^2+^; 11.01 mg/kg for Hg^2+^; 31.33 mg/kg for Cd^2+^; and 129.62 mg/kg for Pb^2+^. EC50 results indicate the ascending toxicity of NB in soil: Cr^6+^ > Cu^2+^ > Ag^+^ > As^3+^ > Ni^2+^ > Hg^2+^ > Cd^2+^ > Pb^2+^. The evaluated toxic elements produced pronounced inhibition of NB O_2_ uptake in soil toxicity tests. The literature consistently notes that toxic elements (Cr, Ni, Pb, As, Cd, Hg) severely hinder AOB and NOB, compromising key nitrification steps [30,31]. These metals interfere with enzymatic activity, membrane integrity, and energy production, leading to reduced ammonia conversion and accumulation of toxic intermediates [32,33]. Cr^6+^ induces oxidative stress and cellular damage before being reduced to Cr^3+^, which further inhibits microbial processes [34,35]. As it disrupts ATP synthesis and enzyme function, Ni, though essential in trace amounts, becomes toxic at higher levels; Cd inactivates enzymes like ammonia monooxygenase; and Hg, highly toxic, damages membranes and DNA, often halting nitrification entirely [36]. Collectively, these disruptions compromise microbial viability and nitrogen cycling, threatening soil fertility and ecosystem health. All reports showed significant decreases in nitrification rates as test-metal levels increased.

### 3.5. Response of Nitrifying Bacteria to Reference Soil and Contaminated Soil

**Figure 5:**
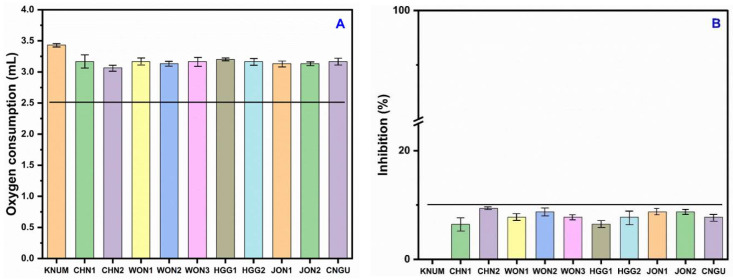
(**A**) Volume of oxygen consumption by NB in the reference soils and (**B**) levels of inhibition of NB in the reference soils in relation to the control KNUM soil.

**Figure 6:**
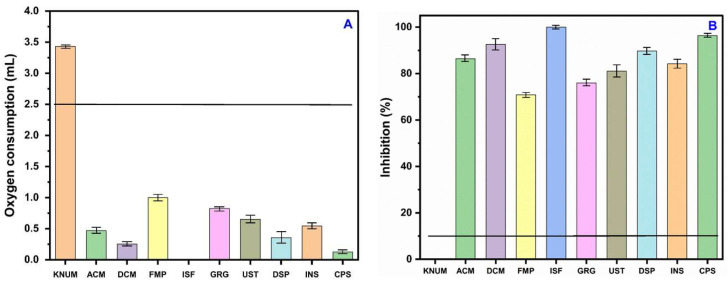
(**A**) Volume of oxygen consumption by NB in the contaminated soils and (**B**) levels of inhibition of NB in the contaminated soils in relation to the control KNUM soil.

The NB-based O_2_ consumption and inhibition results for ten natural environmental samples are presented in Figure 5A,B. The validity criteria for the NB test were satisfied by all ten reference samples, each of which utilized more than 2.5 mL of O_2_. The volume of gas consumed varied between 2.9 mL for HGG 1 and 3.4 mL for KNU M. The validity test criteria were successfully met by all reference soils, which were uncontaminated and contained negligible amounts of heavy metal toxicants. These low levels did not possess the potential to adversely affect O_2_ consumption by NB (Table 1). In contaminated soils, O_2_ consumption ranged from 0.00 mL at ISF to 0.825 mL at GRG; corresponding inhibitions were 71% at FMP, 86% at ACM, and 97% at CPS (Figure 6A,B). The observation of contaminated soils was not surprising, given the extremely high levels of harmful elements recorded—As (143.5–1808.9 mg/kg), Cd (56.4–229.7 mg/kg), Ni (19.4–42.9 mg/kg), Cu (11.5–115.7 mg/kg), and Pb (7.9–408.7 mg/kg)—which have the potential to adversely affect the O_2_ consumption activities of NB. Furthermore, the concentrations far surpassed the EC50 values we obtained for the analyzed metals (see Figure 5 and Figure 6). Recent research indicates that exposure to heavy metals can lead to the downregulation of essential nitrification genes, including amoA, nxrA, and nxrB. This downregulation results in diminished enzymatic activity and reduced oxygen consumption, thereby establishing a connection between the inhibition of functional genes and the noted decrease in nitrifying bacterial respiration [37].

### 3.6. Evidence of Test Validity, Sources of Variability, and Toxicity Thresholds in NB Assays

NB showed minimal inherent test variability in the control soils (Table 4). CVi was 2.15%, marginally higher than [4] 2.1% but lower than the 3.7%, 4.7%, and 2.5% reported for the *Arthrobacter globiformis* solid-contact test [2]. A corresponding value of 3.97% indicated modestly increased variability arising from inherent characteristics of the various reference soils (Table 4). This value also fell below the natural-soil values of 6.8%, 8.9%, 8.1%, and 12.3% reported by [2]. In this study, reference-soil physicochemical properties had no significant impact on NB gas consumption, which explains the relatively low CVi and CVns. The study yielded an MDD of 3.22%. Owing to the substantial variation, we rounded it to the nearest multiple of five to define the MTI as the toxicity threshold. Thus, a 10% value was established as the toxicity threshold for the NB gas-consumption assay. Compared with earlier studies, the 10% toxicity threshold defined in this research renders the NB assay exceptionally sensitive and more reliable for assessing soil toxicity in field conditions than several analogous assays previously employed (Table 5).

**Figure 7:**
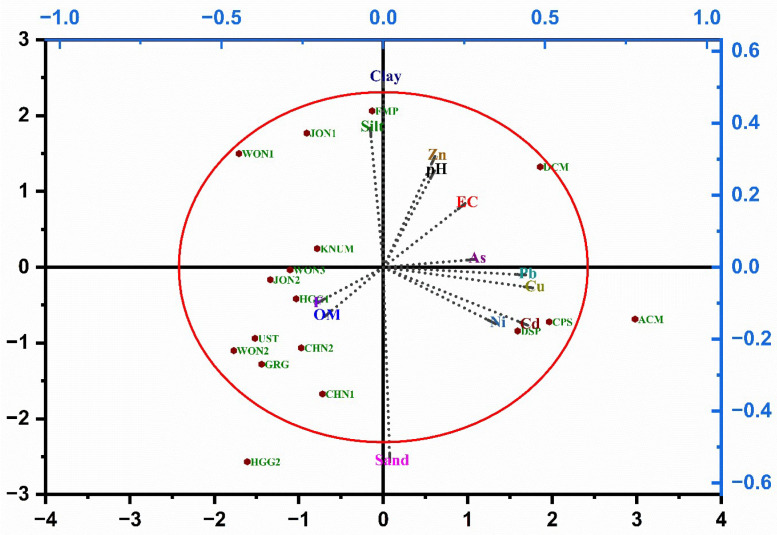
Principal component analysis (PCA) biplot based on the inhibition of oxygen consumption activity of NB by the different physiochemical constituents present in the reference and contaminated soils.

The biplot indicates pronounced variability on Principal Component 1, representing 30.12% of the overall variance. PC1 primarily captured the reference soils, clearly distinguishing them from the rest; their minimal contamination corresponded to high NB gas consumption (Figure 5). PC2 showed less variability (21.1% of the variance) and was occupied by DCM, CPS, DSP, and ACM, which were highly toxic and exceeded the 10% inhibition threshold for NB (Figure 6A,B). As contaminated soils plot along increasing levels of heavy-metal toxicants and thereafter along increasing levels of organic matter in the PCA biplot, it is likely that NB gas-consumption inhibition is chiefly controlled by heavy metals and by the natural properties of the test soils (Figure 7). Figure 3 and Figure 6 demonstrate that heavy metals adversely affect NB gas consumption. As and Zn were positively associated with NB gas consumption in this study (Table 6). This anticipated that inhibition occurred at relatively low concentrations: Ni^2+^ (10.35 mg/kg), As^3+^ (8.99 mg/kg), Cu^2+^ (6.92 mg/kg), Cr^6+^ (1.2 mg/kg), Ag^+^ (8.38 mg), Pb^2+^ (129.62 mg/kg), Hg^2+^ (11.01 mg/kg), and Cd^2+^ (31.33 mg/kg). As the reaction occurred in solution, the acidic conditions of the contaminated soils likely boosted metal bioavailability and, in turn, the toxicity measured in the NB assay. Despite these considerations, the toxicants may act synergistically because most contaminated soils are polluted by combinations of these elements (Table 1).

## 4. Implications, Challenges, and Future Perspectives

The bioassay utilizing nitrifying bacteria (NB) for oxygen consumption, developed in this study, provides a swift, sensitive, and ecologically pertinent method for evaluating soil toxicity resulting from heavy metals and various contaminants, thereby mirroring actual disturbances in microbial nitrogen cycling and soil function. The design of the method, which involves direct contact, along with its low toxicity threshold of 10%, significantly improves its reliability and suitability for field applications compared to traditional elutriate-based assays. Nevertheless, variations in soil composition, organic content, and moisture may influence oxygen consumption, highlighting the importance of further standardization across different soil types. Subsequent research endeavors should prioritize extending the assay to analyze complex contaminant mixtures, integrating molecular biomarkers to enhance mechanistic insights, and developing portable sensor-based field kits for on-site, real-time monitoring.

## 5. Conclusions

This study used an NB direct-contact bioassay to assess reductions in gas consumption and evaluate toxicity in field soils contaminated with harmful elements and natural contaminants. Higher clay concentrations in the test soils significantly influence NB gas consumption. The investigation revealed that the optimal conditions were 1 g of test soil, 1 mL of culture, 10 mL of working medium, 48 h incubation time, and a rotation speed of 100 RPM. For a 50% decrease in O_2_ consumption, the required concentrations were: Cr^6+^ 1.2 mg/kg; Cu^2+^ 6.92 mg/kg; As^3+^ 8.99 mg/kg; Ag^+^ 8.38 mg/kg; Hg^2+^ 11.01 mg/kg; Ni^2+^ 10.35 mg/kg; Cd^2+^ 31.33 mg/kg; Pb^2+^ 129.62 mg/kg. The NB bioassay set the optimal toxicity threshold at 10% for naturally contaminated field soils. A 10% toxicity cutoff renders the NB method highly effective for gauging toxicity in agricultural soils. An important benefit is ease of use: measuring gas consumption with a lubricated glass syringe is straightforward and demands neither specialized training nor equipment. Moreover, the NB gas-consumption test exhibits superior robustness relative to traditional assays, which are frequently impacted by soil physicochemical elements such as clay. When clay is present, fluorescence is often quenched and reduced in spectrophotometric, luminometric, and fluorometric measurements.

## Figures and Tables

**Table 1:** Physicochemical properties of the reference soils (low/non-contaminated soils).

Soil	Land Use	pH	EC (mS/cm)	OM (%)	Sand (%)	Silt (%)	Clay (%)	F (mg/kg)	As (mg/kg)	Cd (mg/kg)	Cu (mg/kg)	Ni (mg/kg)	Pb (mg/kg)	Zn (mg/kg)
KNUM	Mountain	6.17	0.54	5.33	73.13	19.9	6.97	343.3	0	2.35	* ND	* ND	* ND	* ND
CHN1	Upland	7.53	0.44	15.5	95.1	4.2	0.7	371.3	2.5	0.23	3.9	4.37	18.02	3.97
CHN2	Forest	6.47	0.45	33.97	85.2	8.4	6.4	190.3	2.44	0.17	3.17	3.63	18	2.17
WON1	Paddy	5.6	0.42	34.93	73.4	12.4	14.2	432.6	3.43	0.4	9.87	16.43	17.58	3.2
WON2	Urban	7.07	0.23	40.47	80.6	15	4.4	441.3	4.07	0.37	7.57	9.5	14.86	4.37
WON3	Upland	6.67	0.35	22.23	74.9	14.4	10.7	272.3	3.6	0.4	1.43	6.13	17.78	4.27
HGG1	Paddy	6.73	0.48	22.17	79.7	10.2	10.1	466	3.1	0.23	12.17	11.73	12.18	4.77
HGG2	Field	5.67	0.22	26.37	93	3.3	3.7	402.6	4.4	0.3	13.27	6.97	13.63	4.27
JON1	Grassland	7.03	0.20	19.03	77	13.6	9.4	199	8.5	0.43	11	14.1	22.64	2.4
JON2	Upland	6.23	0.29	25.17	72.93	12.8	14.27	386	5.37	0.23	10.13	23.87	15.76	4.33
CNGU	Paddy	6.52	0.40	22.3	45.6	28.0	26.4	287	4.8	0.3	7.0	12.5	15.50	69.0

OM: Organic matter; * ND: Not detected.

**Table 2:** Physicochemical characteristics and contaminant concentrations in polluted soils.

Soil	Land Use	pH	EC (mS/cm)	OM (%)	Sand (%)	Silt (%)	Clay (%)	F (mg/kg)	As (mg/kg)	Cd (mg/kg)	Cu (mg/kg)	Ni (mg/kg)	Pb (mg/kg)	Zn (mg/kg)
ACM	Abandoned Copper	7.37	0.49	11.7	85	9.7	5.3	394.8	1808.9	18.3	79.2	42.9	113.7	295
	Mine													
DCM	Decommissioned	8	0.34	7.17	71.4	19.2	9.3	56	1761.5	2.3	39	19.4	119.3	168
	Coal Mine													
FMP	Fertilizer	7.9	0.6	12.9	64.7	23	12.3	307.6	10	0.64	6.7	3.1	34.5	176
	Manufacturing													
	Plant													
ISF	Iron Smelting	8.1	0.55	24.2	60.7	12.9	26.37	232.4	143.5	7.05	59.8	6.47	275.5	851.9
	Factory													
GRG	Garage	6.2	0.17	28.3	82.6	12.6	4.8	215.8	1.89	2.4	3.1	2.7	7.9	57.7
UST	Underground	6.3	0.12	32.3	78.3	16.4	5.2	194.8	1.9	3.43	2.8	2.7	7.3	63.3
	Storage Tank													
DSP	Decommissioned	6.4	0.26	22	75.5	19	5.5	215.3	352.3	18.8	27.1	27.3	373	18.4
	Sewage Plant													
INS	Incineration Site	6	0.56	26.6	78.3	14.1	7.3	157.9	319.3	28.3	115.7	332.6	408.7	31.5
CPS	Coking Plant Site	7	0.48	13.8	80.9	10.8	8.2	300.7	124.8	12.8	97.8	6.47	200.7	28.4

OM: Organic matter.

**Table 3:** Concentration and test ranges for the tested harmful elements.

Heavy Metal	Test Concentration Range (mg/kg)
Chromium (Cr^6+^)	0.1, 0.5, 1, 2.5, 5, 10, 25
Silver (Ag^+^)	2, 5, 10, 25, 50, 100, 150
Mercury (Hg^2+^)	2, 5, 10, 25, 50, 100, 200
Nickel (Ni^2+^)	5, 10, 25, 50, 100, 150, 250
Arsenic (As^3+^)	2, 5, 10, 25, 50, 100, 150
Copper (Cu^2+^)	2, 5, 10, 20, 50, 100, 200
Cadmium (Cd^2+^)	10, 25, 50, 100, 250, 1000, 2000
Lead (Pb^2+^)	10, 25, 50, 100, 250, 1000, 2000

**Table 4:** Statistical tests used for determining the variability of reference soils and the toxicity threshold.

CV*_i_* (min, max) %	2.12 (1.7, 4.7)
CV*_ns_*	9.11 (7.8, 14.6)
MDD (min, max)	3.22 (2.9, 5.9)
MTI	11.1
Toxicity threshold	13
Number of Reference soils	11

**Table 5:** Comparative analysis of NB soil toxicity test threshold with thresholds obtained using similar bioassays.

Bioassay	Endpoint Parameter	Exposure Time (h)	Toxicity Threshold (%)	References
*Nitrifying bacteria*	Oxygen consumption	48	10	This study
*Chlorella vulgaris*	Oxygen consumption	72	15	[38]
Sulfur-oxidizing bacteria	Oxygen consumption	6	20	[4]
*Anthrobacter*	Dehydrogenase	6	60	[39]
*globitormis*	activity			
*Caenoerhabdtis*	Growth	96	25	
*elegans*	Reproduction	96	50	
*Lumbriculus*	Reproduction	672	25	
*variegatus*				
*Myriophyllum*	Growth	240	20	
*aquaticum*				
*Danio rerio*	Survival	48	20	
*Anthrobacter globiformis*	Dehydrogenase activity	6	45	[2]
*Anthrobacter globiformis*	Dehydrogenase activity	6	40	[40]
*Caenorhabdtis*	Fertility	96	20	[41]
*elegans*	Growth		20	
	Reproduction		40	

**Table 6:** Correlations between oxygen consumption and physiochemical properties on test soils.

	Sand	Slit	Clay	OM	pH	Cd	Zn	Ni	As	F	Cu
GC	−0.5	0.38	0.52	−0.13	0.26	−0.16	0.3	−0.15	0.02	−0.1	−0.05

GC: Gas consumption, OM: Organic matter.

## Data Availability

The original contributions presented in this study are included in the article/Supplementary Materials. Further inquiries can be directed to the corresponding author.

## Supplementary Materials

The following supporting information can be downloaded at: https://www.mdpi.com/article/10.3390/toxics13110937/s1. Figure S1: Profile of the operational parameters monitored in the Nitrifying bacteria master culture reactor (NBMCR); Figure S2: Profile of the operational parameters monitored in the Nitrifying bacteria master culture reactor (NBMCR); Figure S3: Profile of the operational parameters monitored in the nitrifying bacteria master culture reactor (NBMCR); Figure S4: Profile of the operational parameters monitored in the Nitrifying bacteria master culture reactor (NBMCR); Figure S5: Biomass concentration in nitrifying bacteria master culture reactor.
