# Supplementary material for: A Nitrifying Bacteria-Based Oxygen Consumption Assay for Multifaceted Soil Toxicity Monitoring
Source: Toxics. 2025 Oct 30;13(11):937. doi: 10.3390/toxics13110937 (PMC12656603; doi:10.3390/toxics13110937)
Supplement: Supplementary file 1 [file toxics-13-00937-s001.zip › toxics-3910591-supplementary.pdf]

Supporting Information

"A nitrifying bacteria-based oxygen consumption assay for multifaceted soil toxicity monitoring"

Suleman Shahzad[1,a], Aparna Sharma, Syed Ejaz Hussain Mehdi, Fida Hussain, Sandesh Pandey, Woochang Kang[1,a], and Sang Eun Oh[1,*]

[a] Department of Biological Environmental, Kangwon National University Chuncheon-si, Gangwon-do, Hyoja-2-dong, 24341, Republic of Korea

*Corresponding author: Professor Sang Eun Oh

ohsangeun@kangwon.ac.kr

[b] Department of Environmental Engineering, Kwangwoon University, 20 Kwangwoon-Ro, Nowon Gu, Seoul 01897, Republic of Korea

Total pages: 5

Total supporting method: 1

Total results: 4

Total figures: 1

S1 Method 1.

Ion-Chromatography (IC) (Eco IC Metrohm, Swiss). A 5 ml aliquot from the nitrifying bacteria master culture reactor (NBMCR) was treated with sulfuric acid to inhibit alterations in nitrite and nitrate levels throughout the storage period. Centrifuge the mixture at 4000 RPM for 20 minutes at 20°C to facilitate the separation of solid debris from the liquid extract. Carefully remove the supernatant and pass it through a 0.45 μm syringe filter to eliminate any residual particulates. Inject the prepared samples into the chromatographic setup for analysis. The conductivity detector for ion chromatography had a range of 0 to 15,000 μS/cm, while the iPump delivered flow rates from 0.001 to 20 mL/min. Nitrite and nitrate levels in the samples were quantified by analyzing the peak area or height of the respective ions in the chromatogram, relative to established calibration standards (713). The data derived from the chromatograms was examined with attention to any potential dilution factors. The concentrations of nitrite and nitrate in the samples were determined using a calibration curve derived from standard solutions and the magIC net basic software for analysis.

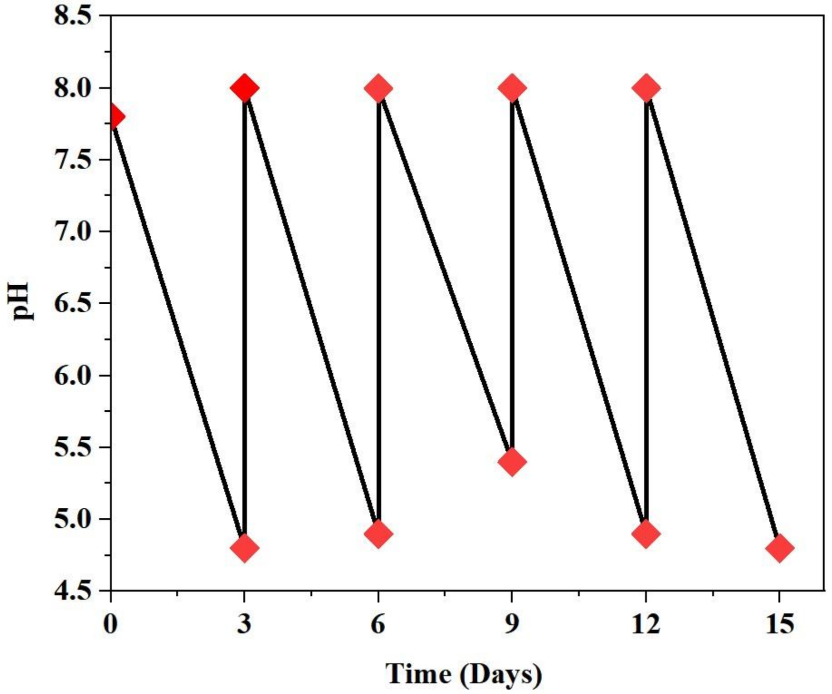

Figure S1. Profile of the operational parameters monitored in the nitrifying bacteria master culture reactor (NBMCR)

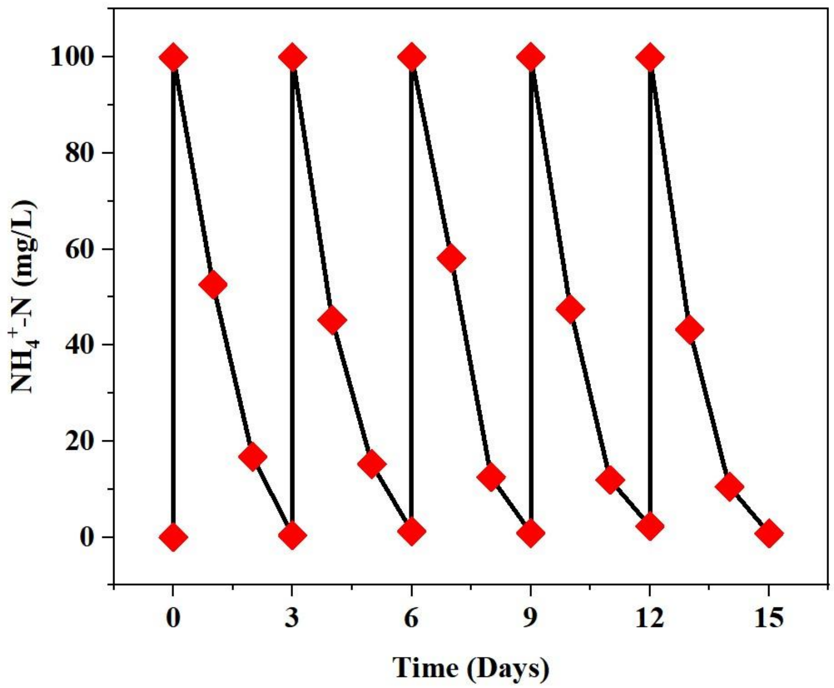

Figure S2. Profile of the operational parameters monitored in the nitrifying bacteria master culture reactor (NBMCR)

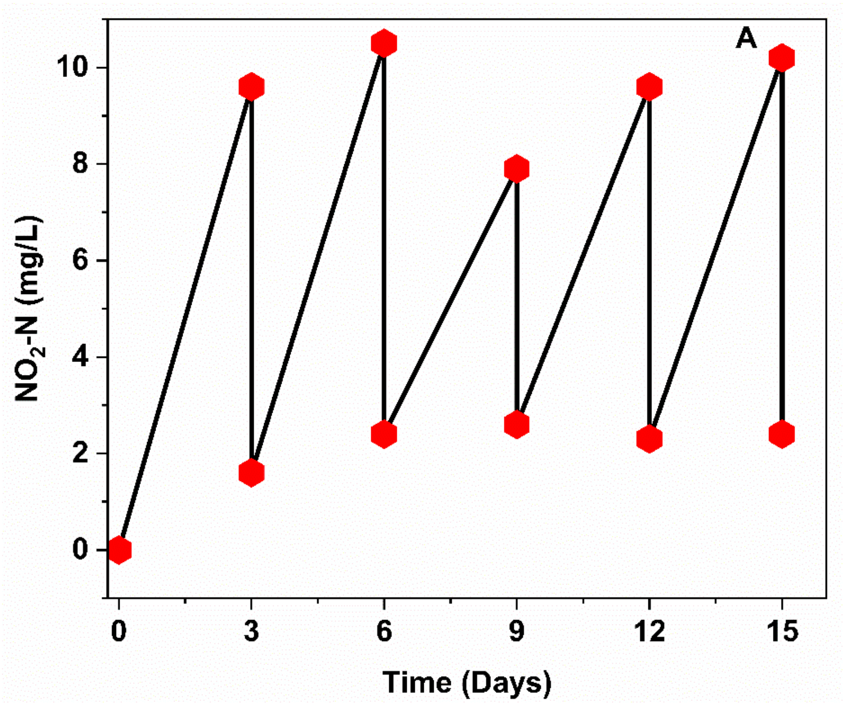

Figure S3. Profile of the operational parameters monitored in the nitrifying bacteria master culture reactor (NBMCR)

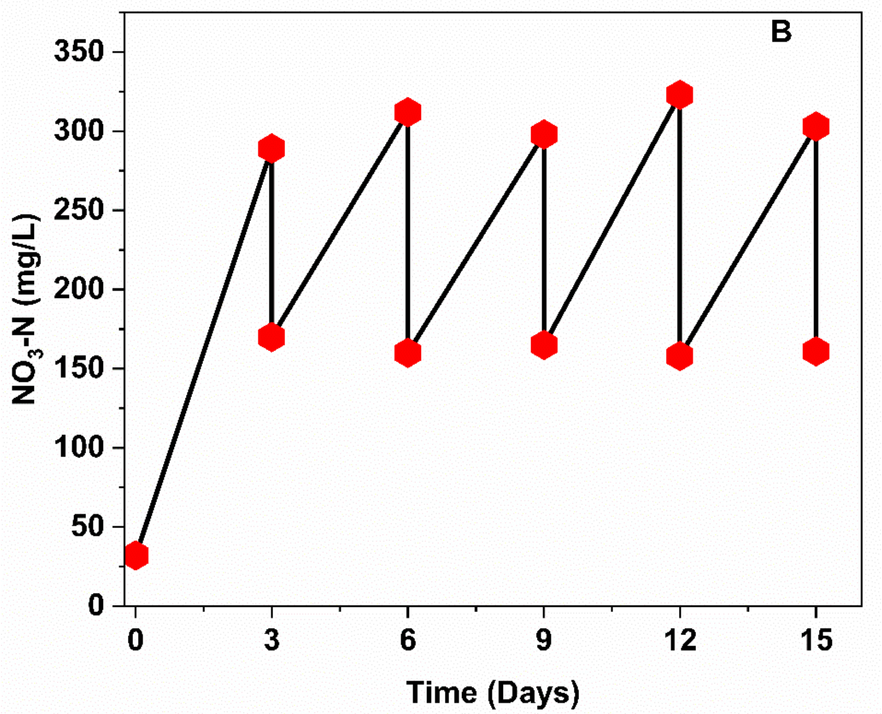

Figure S4. Profile of the operational parameters monitored in the nitrifying bacteria master culture reactor (NBMCR)

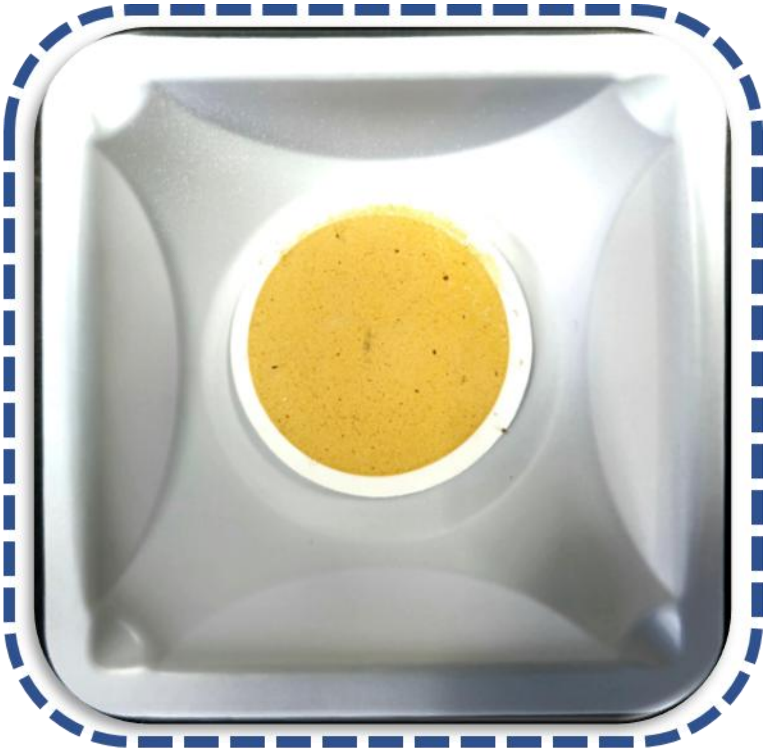

Figure S5 Biomass concentration in the nitrifying bacteria master culture reactor.
